# Review: *In vitro* Cell Platform for Understanding Developmental Toxicity

**DOI:** 10.3389/fgene.2020.623117

**Published:** 2020-12-23

**Authors:** Junkai Xie, Kyle Wettschurack, Chongli Yuan

**Affiliations:** ^1^Davidson School of Chemical Engineering, Purdue University, West Lafayette, IN, United States; ^2^Purdue University Center for Cancer Research, Purdue University, West Lafayette, IN, United States

**Keywords:** IPSC, epigenetics, neurodegenerative disease, developmental exposure, organoid

## Abstract

Developmental toxicity and its affiliation to long-term health, particularly neurodegenerative disease (ND) has attracted significant attentions in recent years. There is, however, a significant gap in current models to track longitudinal changes arising from developmental toxicity. The advent of induced pluripotent stem cell (iPSC) derived neuronal culture has allowed for more complex and functionally active *in vitro* neuronal models. Coupled with recent progress in the detection of ND biomarkers, we are equipped with promising new tools to understand neurotoxicity arising from developmental exposure. This review provides a brief overview of current progress in neuronal culture derived from iPSC and in ND markers.

## Introduction

Developmental exposure to environmental chemicals, such as heavy metal [e.g., lead (Bellinger et al., 1987), manganese (Lynam et al., 1999), mercury (Falluel-Morel et al., 2007)] and organic chemicals (Thiruchelvam et al., 2002) (e.g., pesticides, herbicide, and industrial surfactants) has been associated with various neurodevelopmental and neurodegenerative diseases (NDs) in the past decades and attracted significant attention in the scientific area. These chemicals have pervaded nearly all parts of our environment, including the food chain (Watras et al., 1998), drinking water (Remoundaki et al., 2016), and atmosphere (Lynam et al., 1999) due to decades of industrial and commercial use. Chronic exposure to low-doses of environmental chemicals does not necessarily illicit immediate response in the exposed population but can have significant bearings on shaping the health of individuals later in life (Lee et al., 2003; Wang et al., 2015). Among different exposure windows, the developmental window has been considered the most sensitive to chemical exposure, which has led to the establishment of the Developmental Origins of Health and Disease (DOHaD) hypothesis. The DOHaD hypothesis postulates that the developmental and growth window presents a critical period of time where exposure to certain environmental chemicals can impose significant consequences on an individual’s short and long-term health (Barker, 2007). The attempts to elucidate toxicity arising from developmental exposure, however, have been very challenging because of the latent time between exposure and disease on-set; as well as the lack of established biomarkers that “record” past exposure events and trigger disease on-set later in life.

Longitudinal epidemiology studies have been commonly used to establish the connection between developmental exposure and disease onset later in life. For example, developmental exposure to lead (Pb) can result in an increased risk of developing learning disabilities in exposed children (Needleman et al., 1979) and NDs such as Alzheimer’s disease later in life as suggested in rodent models (Eid et al., 2016). Exposure to organic pesticides such as the herbicide paraquat and fungicide maneb during the developmental stage can cause a decrease of striatal dopamine production and impairment of locomotor activity which aligns with Parkinson’s disease phenotype (Thiruchelvam et al., 2002). Unfortunately, the majority of studies provide limited mechanistic insights into how toxicity affects cells. Various animal models have also been adopted, including rodent (Filipov et al., 2007; Eid et al., 2016), fish (Weber et al., 2013; Wirbisky et al., 2016), and monkey (Lasky et al., 2005) models. These have had success in assessing survival toxicity (Gad, 2014) and damage to reproductive tissues (Song et al., 2014) but have had only limited success for assessing neurotoxicity because of the vast difference between human and non-primate brains. For example, rodent models are extremely popular for the study of cancer (Ding et al., 2011) and psychiatric (Majdak et al., 2016) diseases; however, it has become increasingly clear that rodent models do not necessarily recapitulate human brain function as accurately as previously believed and thus may not be an ideal surrogate system for studying neurotoxicity arising from environmental exposures (Hodge et al., 2019). Although rodent and human brains share a conserved structure, RNA-seq studies have revealed that the expression of ion channels, neurotransmitter synthetase, and neurotransmitter receptors varies greatly between species (Hodge et al., 2019). Uncertainty in the accuracy of animal models for studying brain-related diseases has led to the popularization of human stem cells, including embryonic (ESC) and induced pluripotent stem cells (iPSCs) as an *in vitro* system for assessing neurotoxicity. In contrast to ESC, which must be collected from blastocysts, iPSCs are derived from fibroblasts of human patients. They are easier to collect; account for genetic variations among human patients and thus are becoming a preferred cell model in studying neurotoxicity. iPSCs have been differentiated into various lineages to partially mimic human organs since their debut in 2006 (Takahashi and Yamanaka, 2006). Of particular interest in assessing environmental exposure, iPSCs can be differentiated into neural stem cells (NSCs) (D’Aiuto et al., 2014) and later glutaminergic (Anderson et al., 2015), dopaminergic (Suzuki et al., 2017), and GABAergic (Lin et al., 2015) neurons that mimic part of the brain functions. Here, we will review the recent progress in using human iPSCs as a cell culture model to study neurotoxicity arising from developmental exposure.

## Towards Recaptulating (Part of) Brain Complexity in Human Cell Culture

The brain is a complex organ and thus understandably difficult to reconstruct *in vitro* given its composition, structural, and functional complexity. However, significant progress has been made in recent years enabling partial recapitulation of a human brain in a culture dish.

### Composition Complexity

**FIGURE 1:**
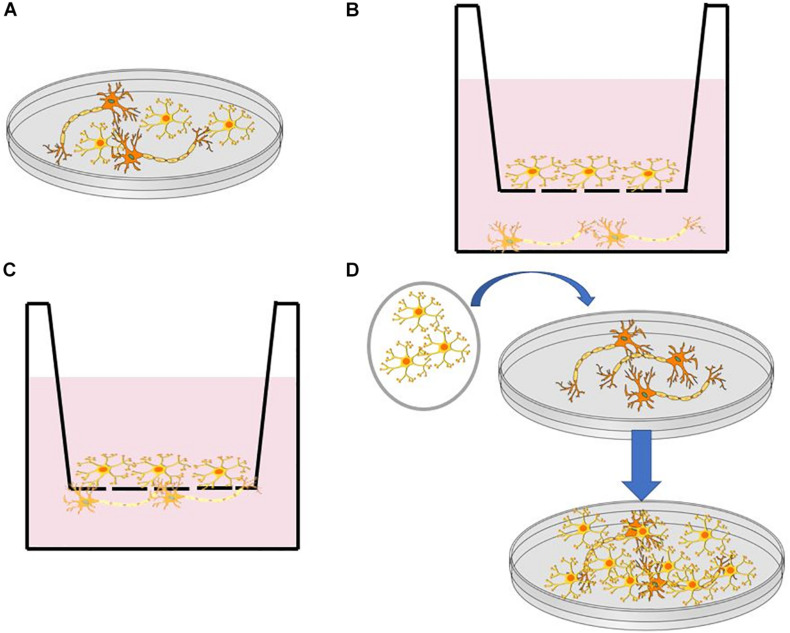
**(A)** Direct co-culture in a dish. **(B)** Indirect co-culture using a transwell insert with a semipermeable membrane. **(C)** Direct co-culture through a semipermeable membrane in a transwell dish. **(D)** Transfer of cell monolayer to established culture through a membrane such as PVDF.

The human brain consists of various cell types, including neurons (Lake et al., 2016), astrocytes (Lewis et al., 2010), and microglia (Hickey and Kimura, 1988). To address the challenge in composition complexity, various protocols have been developed for incorporating multiple cell types via co-culture systems [i.e., neuron- astrocyte (Odawara et al., 2014; Kuijlaars et al., 2016) and neuron- microglia (Haenseler et al., 2017)] differentiated from iPSC and neural progenitor cells (NPCs). Figure 1 illustrates some commonly used co-culturing techniques that can be used to reconstruct brain-like tissues on a dish. These co-culture systems facilitate the reconstruction of complex neural systems resembling neural development (Kuijlaars et al., 2016) or mimicking inflammatory responses that are provoked in NDs such as Alzheimer’s and Parkinson’s disease (McGeer et al., 1988; Sapp et al., 2001). It is worth noting that not all cells found in the brain are from the same stem cell lineage. For example, microglial cells in the brain result from the differentiation of mesodermal progenitors (Murabe and Sano, 1982), rather than the neuroectoderm, as is the case with neurons (Jiang et al., 2003). The combination of multiple types of cells *in vitro* has arisen as a critical tool in the study of brain development, particularly in the study of NDs, where the interaction between multiple cell types is crucial for disease pathology (Di Malta et al., 2012; Heneka et al., 2013).

### Structure Complexity

Significant advances in cell culturing approaches have occurred in recent years that enabled the transition from 2D to 3D cell culture to reconstruct the architecture of the brain. Compared to 3D culture, 2D cell cultures are typically easier to perform and more compatible with imaging-based analysis, including neurite morphology analysis and live-cell tracing (Shin et al., 2018). 3D culture, however, takes advantage of the self-assembly that iPSCs undergo when grown in a 3D environment (Lancaster et al., 2013). This self-assembly causes iPSCs to form distinct cell types native to specific areas of the brain, such as the hippocampus, ventral forebrain, and cerebral cortex (Lancaster et al., 2017; Quadrato et al., 2017). Using this method, researchers can create brain organoids that contain various discrete but interdependent brain regions (Lancaster et al., 2013). This structure complexity, particularly the development and interaction of different brain regions, can produce organoids with active neural networks capable of firing in response to stimuli (Giandomenico et al., 2019). 3D organoids provide an ideal testing platform for drug discovery (Phan et al., 2019), modeling neurological diseases (Lancaster et al., 2013), and assessing environmental toxin exposure (Forsythe et al., 2018). While early brain organoids showed significant heterogeneity (Camp et al., 2015), brain organoids generated via more recent protocols have improved reproducibility (Velasco et al., 2019; Nickels et al., 2020) but still require further optimization.

### Functional Connectivity

Although iPSCs can differentiate into neurons and express the mature neuronal markers such as MAP2 and NeuN, they do not necessarily exhibit the correct electrophysiological properties, hampering their uses in assessing neural circuitry activities. Researchers have developed simplified protocols for differentiating neuronal networks that are electro-physiologically active in 2D (Gunhanlar et al., 2018) and 3D (Paşca et al., 2015; Birey et al., 2017). These protocols typically include cAMP in their culture to facilitate the establishment of neuronal connectivity (Kang et al., 2017; Gunhanlar et al., 2018). With the initial claim of success, the prevalence, composition, properties, and relevance of these established neuronal connections remain to be tested by time. The occurrence of *in vivo*-like complex neuronal activity and functional circuitry remains elusive and more in-depth analysis will be crucial for unraveling the connectivity in cultures, particularly organoids, to understand the impact of genetic and environmental chemical perturbations on human synaptogenesis, neuronal activity, and network function.

## Mimicking Developmental Exposure Using Stem Cell Models

**FIGURE 2:**
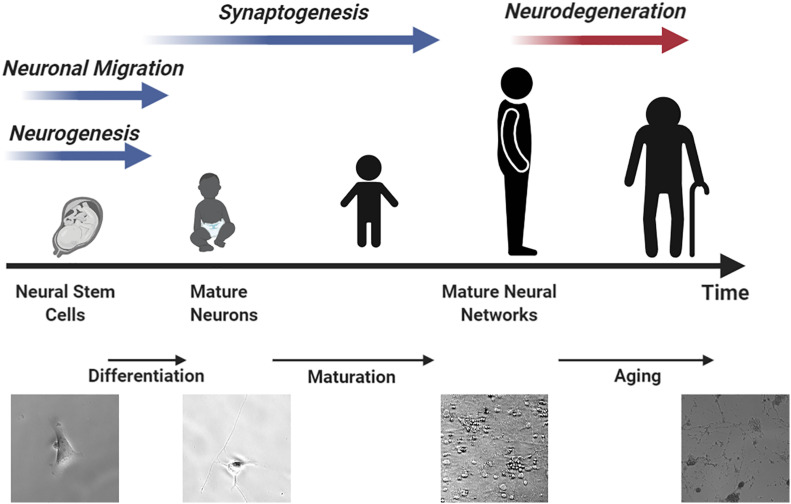
An illustration of neurodevelopment processes matched to different stem cell differentiation time windows resembling human brain development.

Stem cells can undergo differentiation and maturation in a similar manner compared to animal models, providing multiple assessment windows for studying neurotoxicity. Human brains contain many unique features that define cognition (Lui et al., 2011). On average, 86.1 billion neurons can be found in the brain and spinal cord of an adult male and 16.34 billion of these are located in the cerebral cortex (Azevedo et al., 2009; Herculano-Houzel et al., 2016). 80% of the neurons in the cerebral cortex are thought to be excitatory glutaminergic neurons which were differentiated in the ventricular zone and sub-ventricular zone of the cortical wall during prenatal development from 50 postconceptional days (pcd) to 24 postconceptional weeks (pcw) at a rate of ∼3.86 million neurons per hour (Workman et al., 2013). Neurogenesis occurs in the spinal cord and brain stem beginning at 32 pcd followed by cerebral cortex (O’Rahilly, 2006). Most of this neuron generation is completed before birth. Neocortical interneurons and inhibitory GABAergic neurons are two of the few neuron types that are known to undergo differentiation after birth (Sanai et al., 2011; Radonjić et al., 2014). Although most neurons have completed differentiation before birth, the neuronal network is yet to be established. Recent studies have revealed that synaptogenesis (Peter, 1979; Kang et al., 2011), myelination (Miller et al., 2012), and synaptic pruning (Petanjek et al., 2011) are still ongoing until 20 years of age. Figure 2 summarizes the neuronal developmental processes at different time windows in the human brain matched to various stages of stem cell differentiation. Specifically, the procedure of generating mature neurons from NPCs can be divided into two sequential steps, namely, differentiation followed by maturation. During the differentiation step, retinoic acid or bone morphogenetic protein is used to mediate neuronal differentiation signaling pathways (Cazillis et al., 2006). After that, brain-derived neurotrophic factor (BDNF) and dibutyryl cyclic adenosine monophosphate (db-cAMP) are added to facilitate neuron maturation and promote synaptogenesis (Kang et al., 2017; Gunhanlar et al., 2018). The initial differentiation stage is completed after 8 days but it can take up to 6–8 weeks for neurons to mature and develop proper electrophysiological functions (Gunhanlar et al., 2018).

## Assess Neurotoxicity Using Cell Cultur Model

Compared to animal models, cell culture presents several advantages for assessing neurotoxicity, including homogeneity in cell identity, compatibility with long-term monitoring, and controllable dosing. Several disadvantages remain, most notably the inability to perform behavior and aging related assessments. We will thus focus on summarizing molecular markers that have been previously established to facilitate the assessment of neurotoxicity.

### Disease Biomarkers

Cell viability and proliferation are conventionally used as the starting point for assessing neurotoxicity. Although insightful for acute toxicity, limited knowledge can be gained regarding the chronic effects of neurotoxicity arising from environmental exposures. Early-stage disease biomarkers with established correlations of disease on-set thus play a critical role in assessing neurotoxicity. Several good reviews exist summarizing prevalent early-stage biomarkers that have been used to assess ND risks (Molinuevo et al., 2018; Ehrenberg et al., 2020). For example, Aβ42/Aβ40 ratio (Doecke et al., 2020) and phosphorylated tau p-Thr181 tau (Thijssen et al., 2020) and p-Thr205 tau (Barthélemy et al., 2020)) can be used to assist the diagnosis and prognosis of AD. α-synuclein and α-synuclein phosphorylation can be used in PD for similar purposes (Wang et al., 2012; Lin et al., 2017). These biomarkers can be visualized in cells exposed to environmental chemicals using either immunofluorescence or immunohistology methods, or quantified via ELISA, providing a robust approach to connect environmental exposure with neurodevelopmental and NDs.

### Neuronal Activity

Recent expansion of toolsets to conduct electrophysiology measurements have further expanded our capability to probe neural circuit activities related to environmental exposure. Synaptic and ion channel activities, membrane potential, and action potential can be conventionally recorded using patch-clamp and multi-electrode array (MEA). These approaches have been primarily used to record changes in neural circuits of animal brain slices (Cholanian et al., 2017), cultured primary cells (Cannady et al., 2017; Dunn et al., 2018), and cell cultures (James et al., 2017) responding to various environmental chemical exposures. Neurons derived from iPSCs can establish electro-physiologically mature neuronal networks that closely resemble mature primary neurons (Gunhanlar et al., 2018). Recently, patch-clamp and MEA have been applied to cortical neurons derived from iPSCs of AD patient and healthy control; and demonstrated that AD neuronal cultures exhibit increased spontaneous firing, slow oscillatory events, and hypersynchronous circuit activity (Ghatak et al., 2020). Furthermore, various imaging probes, including calcium indicators, i.e., Fura-2 (Tsien et al., 1982; Grynkiewicz et al., 1985) and GCaMP (Chen et al., 2013; Yang et al., 2018); membrane potential probes, i.e., ASAP2 (Yang et al., 2016; Chamberland et al., 2017) and Voltron (Abdelfattah et al., 2019); and neurotransmitter probes, i.e., iGluSnFR (Marvin et al., 2013) and dLight (Patriarchi et al., 2018) can be introduced to neuronal cultures to monitor neural circuit activity *in situ*. These measurements collectively provide a viable approach to characterize abnormal circuitry activity that are known to be altered in various neurodevelopmental and neurodegenerative conditions. For example, AD neuronal cultures show oscillatory events and hypersynchronous network activity compared to their wild-type isogenic controls (Ghatak et al., 2020). Furthermore, neurons derived from iPSCs of Autism Spectrum Disorders (ASD) have significantly altered glutamate neurotransmitter release and reduced spontaneous firing rate (Russo et al., 2018).

### Epigenetic Changes

Epigenetic modifications account for inheritable changes in chromatin that are not accounted for by DNA mutations. Epigenetic modifications have been increasingly recognized as viable markers for various neurological diseases and are being actively researched. Among different epigenetic modifications, DNA methylation, such as cytosine methylation (5mC), hydroxymethylation (5hmC), and histone acetylation have attracted the most significant attention. Mutations in DNA and histone methyltransferase have been identified as risk factors in autism (Satterstrom et al., 2020). In addition, abnormal alterations in H3K9ac and H3K27ac were identified as the major distinctions between aging and AD brains (Nativio et al., 2020). Furthermore, CpH hypomethylation is accelerated in AD brains compared to normal aging ones (Li et al., 2019). Abnormal DNA methylation of ASCC1 and SLC7A11 has also been linked to PD (Vallerga et al., 2020). Similar methylation features were also revealed in iPSC models reprogrammed from patients iPSCs (Fetahu et al., 2019). Interestingly, environmental exposure of neuronal cells can also lead to major changes in DNA methylation, histone methylation, and acetylation. This suggests that epigenetic mechanisms may serve as a potential bridging marker between environmental exposure and neurological conditions (Pavanello et al., 2009; Luo et al., 2014; Wirbisky-Hershberger et al., 2017; Lin et al., 2020). The detection of epigenetic changes by protein-based fluorescent probes allows for real-time analysis of the changes to the global epigenome levels. So far protein probes have been developed for binding and visualization of both DNA methylation and histone modifications (Hendrich and Bird, 1998; Lungu et al., 2017; Sanchez et al., 2017; Sanchez et al., 2019). Further development of nanobody fragments have provided an additional domain that allows for high specificity and affinity for selected epigenetic targets (Hattori et al., 2016; Jullien et al., 2016). Accurate understanding and quantification of epigenetic changes that proceed chemically induced disease development could provide a system that can detect neurological conditions at an earlier time point.

## Conclusion

In closing, patient derived iPSCs offer a promising platform to assess neurotoxicity given its ability to partially recapitulate the complexity and functionality of *in vivo* neurological structures. The advent and exploration of both 2D and 3D iPSC systems provide great promise for the examination of environmental exposure effects on adolescent brains. In addition, these cell assemblies provide a platform that more closely matches the developmental timeline and disease phenotypes of a human brain in comparison to rodent models. The application of environmental chemicals to iPSC is in its early stages, but provides a clear opportunity to understand the mechanisms that affect the human brain when exposed to heavy metals and pesticides. The use of phenotypic assays that quantify ND risk factors, neuron activity, and epigenetic changes can be used to elucidate the molecular mechanisms that are perturbed by environmental exposure. With the application of the numerous live-cell compatible techniques to human iPSCs, we are at an optimal position to understand the biological response to chronic toxin exposure and how it informs long-term disease development.

## Author Contributions

All authors have contributed in planning and writing the review.

## Conflict of Interest

The authors declare that the research was conducted in the absence of any commercial or financial relationships that could be construed as a potential conflict of interest.

## References

[B1] Abdelfattah A. S. Kawashima T. Singh A. Novak O. Liu H. Shuai Y. (2019). Bright and photostable chemigenetic indicators for extended in vivo voltage imaging. *Science* 365 699–704. 10.1126/science.aav6416 31371562

[B2] Anderson G. W. Deans P. J. M. Taylor R. D. T. Raval P. Chen D. Lowder H. (2015). Characterisation of neurons derived from a cortical human neural stem cell line CTX0E16. *Stem Cell Res. Ther.* 6:149. 10.1186/s13287-015-0136-8 PMC4546258 26296747

[B3] Azevedo F. A. C. Carvalho L. R. B. Grinberg L. T. Farfel J. M. Ferretti R. E. L. Leite R. E. P. (2009). Equal numbers of neuronal and nonneuronal cells make the human brain an isometrically scaled-up primate brain. *J. Comp. Neurol.* 513 532–541. 10.1002/cne.21974 19226510

[B4] Barker D. J. (2007). The origins of the developmental origins theory. *J. Intern. Med.* 261 412–417. 10.1111/j.1365-2796.2007.01809.x 17444880

[B5] Barthélemy N. R. Li Y. Joseph-Mathurin N. Gordon B. A. Hassenstab J. Benzinger T. L. S. (2020). A soluble phosphorylated tau signature links tau., amyloid and the evolution of stages of dominantly inherited Alzheimer’s disease. *Nat. Med.* 26 398–407. 10.1038/s41591-020-0781-z 32161412 PMC7309367

[B6] Bellinger D. Leviton A. Waternaux C. Needleman H. Rabinowitz M. (1987). Longitudinal analyses of prenatal and postnatal lead exposure and early cognitive development. *N. Engl. J. Med.* 316 1037–1043. 10.1056/nejm198704233161701 3561456

[B7] Birey F. Andersen J. Makinson C. D. Islam S. Wei W. Huber N. (2017). Assembly of functionally integrated human forebrain spheroids. *Nature* 545 54–59. 10.1038/nature22330 28445465 PMC5805137

[B8] Camp J. G. Badsha F. Florio M. Kanton S. Gerber T. Wilsch-Bräuninger M. (2015). Human cerebral organoids recapitulate gene expression programs of fetal neocortex development. *Proc. Natl. Acad. Sci. U.S.A.* 112 15672–15677. 10.1073/pnas.1520760112 26644564 PMC4697386

[B9] Cannady R. McGonigal J. T. Newsom R. J. Woodward J. J. Mulholland P. J. Gass J. T. (2017). Prefrontal Cortex K(Ca)2 channels regulate mGlu(5)-Dependent plasticity and extinction of alcohol-seeking behavior. *J. Neurosci.* 37 4359–4369. 10.1523/jneurosci.2873-16.2017 28320841 PMC5413180

[B10] Cazillis M. Rasika S. Mani S. Gressens P. Leliévre V. (2006). In vitro induction of neural differentiation of embryonic stem (ES) cells closely mimics molecular mechanisms of embryonic brain development. *Pediatr. Res.* 59 48–53. 10.1203/01.pdr.0000203566.01600.8c 16549548

[B11] Chamberland S. Yang H. H. Pan M. M. Evans S. W. Guan S. Chavarha M. (2017). Fast two-photon imaging of subcellular voltage dynamics in neuronal tissue with genetically encoded indicators. *eLife* 6:e25690. 10.7554/eLife.25690 PMC5584994 28749338

[B12] Chen T.-W. Wardill T. J. Sun Y. Pulver S. R. Renninger S. L. Baohan A. (2013). Ultrasensitive fluorescent proteins for imaging neuronal activity. *Nature* 499 295–300. 10.1038/nature12354 23868258 PMC3777791

[B13] Cholanian M. Wealing J. Levine R. B. Fregosi R. F. (2017). Developmental nicotine exposure alters potassium currents in hypoglossal motoneurons of neonatal rat. *J. Neurophysiol.* 117 1544–1552. 10.1152/jn.00774.2016 28148643 PMC5376599

[B14] D’Aiuto L. Zhi Y. Kumar Das D. Wilcox M. R. Johnson J. W. McClain L. (2014). Large-scale generation of human iPSC-derived neural stem cells/early neural progenitor cells and their neuronal differentiation. *Organogenesis* 10 365–377. 10.1080/15476278.2015.1011921 25629202 PMC4594592

[B15] Di Malta C. Fryer J. D. Settembre C. Ballabio A. (2012). Astrocyte dysfunction triggers neurodegeneration in a lysosomal storage disorder. *Proc. Natl. Acad. Sci. U.S.A.* 109 E2334–E2342. 22826245 10.1073/pnas.1209577109 PMC3435187

[B16] Ding Z. Wu C.-J. Chu G. C. Xiao Y. Ho D. Zhang J. (2011). SMAD4-dependent barrier constrains prostate cancer growth and metastatic progression. *Nature* 470 269–273. 10.1038/nature09677 21289624 PMC3753179

[B17] Doecke J. D. Pérez-Grijalba V. Fandos N. Fowler C. Villemagne V. L. Masters C. L. (2020). Total Aβ42/Aβ40 ratio in plasma predicts amyloid-PET status, independent of clinical AD diagnosis. *Neurology* 94 e1580–e1591. 32179698 10.1212/WNL.0000000000009240 PMC7251518

[B18] Dunn A. R. Neuner S. M. Ding S. Hope K. A. O’Connell K. M. S. Kaczorowski C. C. (2018). Cell-type-specific changes in intrinsic excitability in the subiculum following learning and exposure to novel environmental contexts. *eNeuro* 5:ENEURO.0484-18.2018. 10.1523/ENEURO.0484-18.2018 30627661 PMC6325565

[B19] Ehrenberg A. J. Khatun A. Coomans E. Betts M. J. Capraro F. Thijssen E. H. (2020). Relevance of biomarkers across different neurodegenerative diseases. *Alzheimers Res. Ther.* 13:56. 10.1186/s13195-020-00601-w PMC7222479 32404143

[B20] Eid A. Bihaqi S. W. Renehan W. E. Zawia N. H. (2016). Developmental lead exposure and lifespan alterations in epigenetic regulators and their correspondence to biomarkers of Alzheimer’s disease. *Alzheimer’s Dement.* 2 123–131. 10.1016/j.dadm.2016.02.002 27239543 PMC4879653

[B21] Falluel-Morel A. Sokolowski K. Sisti H. M. Zhou X. Shors T. J. DiCicco-Bloom E. (2007). Developmental mercury exposure elicits acute hippocampal cell death., reductions in neurogenesis., and severe learning deficits during puberty. *J. Neurochem.* 103 1968–1981. 10.1111/j.1471-4159.2007.04882.x 17760861 PMC3363963

[B22] Fetahu I. S. Ma D. Rabidou K. Argueta C. Smith M. Liu H. (2019). Epigenetic signatures of methylated DNA cytosine in Alzheimer’s disease. *Sci. Adv.* 5:eaaw2880. 10.1126/sciadv.aaw2880 31489368 PMC6713504

[B23] Filipov N. M. Stewart M. A. Carr R. L. Sistrunk S. C. (2007). Dopaminergic toxicity of the herbicide atrazine in rat striatal slices. *Toxicology* 232 68–78. 10.1016/j.tox.2006.12.007 17218051 PMC1853311

[B24] Forsythe S. D. Devarasetty M. Shupe T. Bishop C. Atala A. Soker S. (2018). Environmental toxin screening using human-derived 3d bioengineered liver and cardiac organoids. *Front. Public Health* 6:103. 10.3389/fpubh.2018.00103 29755963 PMC5932352

[B25] Gad S. C. (2014). “Chapter 2 - Rodents model for toxicity testing and biomarkers,” in *Biomarkers in Toxicology*, ed. Gupta R. C. (Boston, MA: Academic Press), 7–69. 10.1016/b978-0-12-404630-6.00002-6

[B26] Ghatak S. Dolatabadi N. Gao R. Wu Y. Scott H. Trudler D. (2020). NitroSynapsin ameliorates hypersynchronous neural network activity in Alzheimer hiPSC models. *Mol. Psychiatry* 10.1038/s41380-020-0776-7 [Epub ahead of print]. 32467645 PMC7704704

[B27] Giandomenico S. L. Mierau S. B. Gibbons G. M. Wenger L. M. D. Masullo L. Sit T. (2019). Cerebral organoids at the air–liquid interface generate diverse nerve tracts with functional output. *Nat. Neurosci.* 22 669–679. 10.1038/s41593-019-0350-2 30886407 PMC6436729

[B28] Grynkiewicz G. Poenie M. Tsien R. Y. (1985). A new generation of Ca2+ indicators with greatly improved fluorescence properties. *J. Biol. Chem.* 260 3440–3450. 3838314

[B29] Gunhanlar N. Shpak G. van der Kroeg M. Gouty-Colomer L. A. Munshi S. T. Lendemeijer B. (2018). A simplified protocol for differentiation of electrophysiologically mature neuronal networks from human induced pluripotent stem cells. *Mol. Psychiatry* 23 1336–1344. 10.1038/mp.2017.56 28416807 PMC5984104

[B30] Haenseler W. Sansom S. N. Buchrieser J. Newey S. E. Moore C. S. Nicholls F. J. (2017). A highly efficient human pluripotent stem cell microglia model displays a neuronal-co-culture-specific expression profile and inflammatory response. *Stem Cell Rep.* 8 1727–1742. 10.1016/j.stemcr.2017.05.017 28591653 PMC5470330

[B31] Hattori T. Lai D. Dementieva I. S. Montano S. P. Kurosawa K. Zheng Y. P. (2016). Antigen clasping by two antigen-binding sites of an exceptionally specific antibody for histone methylation. *Proc. Natl. Acad. Sci. U.S.A.* 113 2092–2097. 10.1073/pnas.1522691113 26862167 PMC4776465

[B32] Hendrich B. Bird A. (1998). Identification and characterization of a family of mammalian methyl-CpG binding proteins. *Mol. Cell. Biol.* 18 6538–6547. 10.1128/mcb.18.11.6538 9774669 PMC109239

[B33] Heneka M. T. Kummer M. P. Stutz A. Delekate A. Schwartz S. Vieira-Saecker A. (2013). NLRP3 is activated in Alzheimer’s disease and contributes to pathology in APP/PS1 mice. *Nature* 493 674–678. 10.1038/nature11729 23254930 PMC3812809

[B34] Herculano-Houzel S. Kaas J. H. de Oliveira-Souza R. (2016). Corticalization of motor control in humans is a consequence of brain scaling in primate evolution. *J. Comp. Neurol.* 524 448–455. 10.1002/cne.23792 25891512

[B35] Hickey W. F. Kimura H. (1988). Perivascular microglial cells of the CNS are bone marrow-derived and present antigen in vivo. *Science* 239 290–292. 10.1126/science.3276004 3276004

[B36] Hodge R. D. Bakken T. E. Miller J. A. Smith K. A. Barkan E. R. Graybuck L. T. (2019). Conserved cell types with divergent features in human versus mouse cortex. *Nature* 573 61–68. 31435019 10.1038/s41586-019-1506-7 PMC6919571

[B37] James T. F. Nenov M. N. Tapia C. M. Lecchi M. Koshy S. Green T. A. (2017). Consequences of acute Na(v)1.1 exposure to deltamethrin. *Neurotoxicology* 60 150–160. 10.1016/j.neuro.2016.12.005 28007400 PMC5447465

[B38] Jiang Y. Henderson D. Blackstad M. Chen A. Miller R. F. Verfaillie C. M. (2003). Neuroectodermal differentiation from mouse multipotent adult progenitor cells. *Proc. Natl. Acad. Sci. U.S.A.* 100 11854–11860. 10.1073/pnas.1834196100 12925733 PMC304098

[B39] Jullien D. Vignard J. Fedor Y. Bery N. Olichon A. Crozatier M. (2016). Chromatibody., a novel non-invasive molecular tool to explore and manipulate chromatin in living cells. *J. Cell Sci.* 129 2673–2683. 10.1242/jcs.183103 27206857 PMC4958301

[B40] Kang H. J. Kawasawa Y. I. Cheng F. Zhu Y. Xu X. Li M. (2011). Spatio-temporal transcriptome of the human brain. *Nature* 478 483–489. 22031440 10.1038/nature10523 PMC3566780

[B41] Kang S. Chen X. Gong S. Yu P. Yau S. Su Z. (2017). Characteristic analyses of a neural differentiation model from iPSC-derived neuron according to morphology., physiology., and global gene expression pattern. *Sci. Rep.* 7:12233. 10.1038/s41598-017-12452-x PMC5612987 28947763

[B42] Kuijlaars J. Oyelami T. Diels A. Rohrbacher J. Versweyveld S. Meneghello G. (2016). Sustained synchronized neuronal network activity in a human astrocyte co-culture system. *Sci. Rep.* 6:36529. 10.1038/srep36529 PMC5098163 27819315

[B43] Lake B. B. Ai R. Kaeser G. E. Salathia N. S. Yung Y. C. Liu R. (2016). Neuronal subtypes and diversity revealed by single-nucleus RNA sequencing of the human brain. *Science* 352 1586–1590. 10.1126/science.aaf1204 27339989 PMC5038589

[B44] Lancaster M. A. Corsini N. S. Wolfinger S. Gustafson E. H. Phillips A. W. Burkard T. R. (2017). Guided self-organization and cortical plate formation in human brain organoids. *Nat. Biotechnol.* 35 659–666. 10.1038/nbt.3906 28562594 PMC5824977

[B45] Lancaster M. A. Renner M. Martin C.-A. Wenzel D. Bicknell L. S. Hurles M. E. (2013). Cerebral organoids model human brain development and microcephaly. *Nature* 501 373–379. 23995685 10.1038/nature12517 PMC3817409

[B46] Lasky R. E. Luck M. L. Parikh N. A. Laughlin N. K. (2005). The effects of early lead exposure on the brains of adult rhesus monkeys: a volumetric MRI study. *Toxicol. Sci.* 85 963–975. 10.1093/toxsci/kfi153 15788724

[B47] Lee Y. L. Pai M. C. Chen J. H. Guo Y. L. (2003). Central neurological abnormalities and multiple chemical sensitivity caused by chronic toluene exposure. *Occup. Med.* 53 479–482. 10.1093/occmed/kqg095 14581647

[B48] Lewis N. E. Schramm G. Bordbar A. Schellenberger J. Andersen M. P. Cheng J. K. (2010). Large-scale in silico modeling of metabolic interactions between cell types in the human brain. *Nat. Biotechnol.* 28 1279–1285. 10.1038/nbt.1711 21102456 PMC3140076

[B49] Li P. Marshall L. Oh G. Jakubowski J. L. Groot D. He Y. (2019). Epigenetic dysregulation of enhancers in neurons is associated with Alzheimer’s disease pathology and cognitive symptoms. *Nat. Commun.* 10:2246. 10.1038/s41467-019-10101-7 PMC6529540 31113950

[B50] Lin C.-H. Yang S.-Y. Horng H.-E. Yang C.-C. Chieh J.-J. Chen H.-H. (2017). Plasma α-synuclein predicts cognitive decline in Parkinson’s disease. *J. Neurol.Neurosurg. Psychiatry* 88 818–824. 10.1136/jnnp-2016-314857 28550072 PMC5629933

[B51] Lin L. Xie J. Sanchez O. F. Bryan C. Freeman J. Yuan C. (2020). Low dose lead exposure induces alterations on heterochromatin hallmarks persisting through SH-SY5Y cell differentiation. *Chemosphere* 264:128486. 10.1016/j.chemosphere.2020.128486 33032221

[B52] Lin L. Yuan J. Sander B. Golas M. M. (2015). In vitro differentiation of human neural progenitor cells into striatal GABAergic neurons. *Stem Cells Transl. Med.* 4 775–788. 10.5966/sctm.2014-0083 25972145 PMC4479615

[B53] Lui J. H. Hansen D. V. Kriegstein A. R. (2011). Development and evolution of the human neocortex. *Cell* 146 18–36. 10.1016/j.cell.2011.06.030 21729779 PMC3610574

[B54] Lungu C. Pinter S. Broche J. Rathert P. Jeltsch A. (2017). Modular fluorescence complementation sensors for live cell detection of epigenetic signals at endogenous genomic sites. *Nat. Commun.* 8:649. 10.1038/s41467-017-00457-z PMC5608954 28935858

[B55] Luo M. Xu Y. Cai R. Tang Y. Ge M.-M. Liu Z.-H. (2014). Epigenetic histone modification regulates developmental lead exposure induced hyperactivity in rats. *Toxicol. Lett.* 225 78–85. 10.1016/j.toxlet.2013.11.025 24291742

[B56] Lynam D. R. Roos J. W. Pfeifer G. D. Fort B. F. Pullin T. G. (1999). Environmental effects and exposures to manganese from use of methylcyclopentadienyl manganese tricarbonyl (MMT) in gasoline. *Neurotoxicology* 20 145–150. 10385878

[B57] Majdak P. Ossyra J. R. Ossyra J. M. Cobert A. J. Hofmann G. C. Tse S. (2016). A new mouse model of ADHD for medication development. *Sci. Rep.* 6:39472. 10.1038/srep39472 PMC5171883 27996970

[B58] Marvin J. S. Borghuis B. G. Tian L. Cichon J. Harnett M. T. Akerboom J. (2013). An optimized fluorescent probe for visualizing glutamate neurotransmission. *Nat. Methods* 10 162–170. 10.1038/nmeth.2333 23314171 PMC4469972

[B59] McGeer P. L. Itagaki S. Boyes B. E. McGeer E. G. (1988). Reactive microglia are positive for HLA-DR in the substantia nigra of Parkinson’s and Alzheimer’s disease brains. *Neurology* 38 1285–1285. 10.1212/wnl.38.8.1285 3399080

[B60] Miller D. J. Duka T. Stimpson C. D. Schapiro S. J. Baze W. B. McArthur M. J. (2012). Prolonged myelination in human neocortical evolution. *Proc. Natl. Acad. Sci. U.S.A.* 109 16480–16485. 10.1073/pnas.1117943109 23012402 PMC3478650

[B61] Molinuevo J. L. Ayton S. Batrla R. Bednar M. M. Bittner T. Cummings J. (2018). Current state of Alzheimer’s fluid biomarkers. *Acta Neuropathol.* 136 821–853. 30488277 10.1007/s00401-018-1932-x PMC6280827

[B62] Murabe Y. Sano Y. (1982). Morphological studies on neuroglia. *Cell Tissue Res.* 225 469–485. 6290069 10.1007/BF00214798

[B63] Nativio R. Lan Y. Donahue G. Sidoli S. Berson A. Srinivasan A. R. (2020). An integrated multi-omics approach identifies epigenetic alterations associated with Alzheimer’s disease. *Nat. Genet.* 52 1024–1035. 10.1038/s41588-020-0696-0 32989324 PMC8098004

[B64] Needleman H. L. Gunnoe C. Leviton A. Reed R. Peresie H. Maher C. (1979). Deficits in psychologic and classroom performance of children with elevated dentine lead levels. *N. Engl. J. Med.* 300 689–695. 10.1056/nejm197903293001301 763299

[B65] Nickels S. L. Modamio J. Mendes-Pinheiro B. Monzel A. S. Betsou F. Schwamborn J. C. (2020). Reproducible generation of human midbrain organoids for in vitro modeling of Parkinson’s disease. *Stem Cell Res.* 46:101870. 10.1016/j.scr.2020.101870 32534166

[B66] Odawara A. Saitoh Y. Alhebshi A. H. Gotoh M. Suzuki I. (2014). Long-term electrophysiological activity and pharmacological response of a human induced pluripotent stem cell-derived neuron and astrocyte co-culture. *Biochem. Biophys. Res. Commun.* 443 1176–1181. 10.1016/j.bbrc.2013.12.142 24406164

[B67] O’Rahilly F. M. (2006). “Embryonic Staging,” in *The Embryonic Human Brain: An Atlas of Developmental Stages*, 3rd Edn, ed. John Wiley & Sons (Hoboken, NJ: Wiley), 11–13. 10.1002/0471973084.ch4

[B68] Paşca A. M. Sloan S. A. Clarke L. E. Tian Y. Makinson C. D. Huber N. (2015). Functional cortical neurons and astrocytes from human pluripotent stem cells in 3D culture. *Nat. Methods* 12 671–678. 10.1038/nmeth.3415 26005811 PMC4489980

[B69] Patriarchi T. Cho J. R. Merten K. Howe M. W. Marley A. Xiong W.-H. (2018). Ultrafast neuronal imaging of dopamine dynamics with designed genetically encoded sensors. *Science* 360:eaat4422. 10.1126/science.aat4422 29853555 PMC6287765

[B70] Pavanello S. Bollati V. Pesatori A. C. Kapka L. Bolognesi C. Bertazzi P. A. (2009). Global and gene-specific promoter methylation changes are related to anti-B [a] PDE-DNA adduct levels and influence micronuclei levels in polycyclic aromatic hydrocarbon-exposed individuals. *Int. J. Cancer* 125 1692–1697. 10.1002/ijc.24492 19521983

[B71] Petanjek Z. Judaš M. Šimić G. Rašin M. R. Uylings H. B. M. Rakic P. (2011). Extraordinary neoteny of synaptic spines in the human prefrontal cortex. *Proc. Natl. Acad. Sci. U.S.A.* 108 13281–13286. 10.1073/pnas.1105108108 21788513 PMC3156171

[B72] Peter R. H. (1979). Synaptic density in human frontal cortex — Developmental changes and effects of aging. *Brain Res.* 163 195–205. 10.1016/0006-8993(79)90349-4 427544

[B73] Phan N. Hong J. J. Tofig B. Mapua M. Elashoff D. Moatamed N. A. (2019). A simple high-throughput approach identifies actionable drug sensitivities in patient-derived tumor organoids. *Commun. Biol.* 2:78. 10.1038/s42003-019-0305-x PMC6389967 30820473

[B74] Quadrato G. Nguyen T. Macosko E. Z. Sherwood J. L. Min Yang S. Berger D. R. (2017). Cell diversity and network dynamics in photosensitive human brain organoids. *Nature* 545 48–53. 10.1038/nature22047 28445462 PMC5659341

[B75] Radonjić N. V. Ayoub Albert E. Memi F. Yu X. Maroof A. Jakovcevski I. (2014). Diversity of cortical interneurons in primates: the role of the dorsal proliferative niche. *Cell Rep.* 9 2139–2151. 10.1016/j.celrep.2014.11.026 25497090 PMC4306459

[B76] Remoundaki E. Vasileiou E. Philippou A. Perraki M. Kousi P. Hatzikioseyian A. (2016). Groundwater deterioration: the simultaneous effects of intense agricultural activity and heavy metals in soil. *Proc. Eng.* 162 545–552. 10.1016/j.proeng.2016.11.099

[B77] Russo F. B. Freitas B. C. Pignatari G. C. Fernandes I. R. Sebat J. Muotri A. R. (2018). Modeling the interplay between neurons and astrocytes in autism using human induced pluripotent stem cells. *Biol. Psychiatry* 83 569–578. 10.1016/j.biopsych.2017.09.021 29129319

[B78] Sanai N. Nguyen T. Ihrie R. A. Mirzadeh Z. Tsai H.-H. Wong M. (2011). Corridors of migrating neurons in the human brain and their decline during infancy. *Nature* 478 382–386. 10.1038/nature10487 21964341 PMC3197903

[B79] Sanchez O. F. Mendonca A. Carneiro A. D. Yuan C. L. (2017). Engineering recombinant protein sensors for quantifying histone acetylation. *ACS Sens.* 2 426–435. 10.1021/acssensors.7b00026 28723212

[B80] Sanchez O. F. Mendonca A. Min A. Liu J. C. Yuan C. L. (2019). Monitoring histone methylation (H3K9me3) changes in live cells. *ACS Omega* 4 13250–13259. 10.1021/acsomega.9b01413 31460452 PMC6705211

[B81] Sapp E. Kegel K. B. Aronin N. Hashikawa T. Uchiyama Y. Tohyama K. (2001). Early and progressive accumulation of reactive microglia in the Huntington disease brain. *J. Neuropathol. Exp. Neurol.* 60 161–172. 10.1093/jnen/60.2.161 11273004

[B82] Satterstrom F. K. Kosmicki J. A. Wang J. Breen M. S. De Rubeis S. An J. Y. (2020). Large-scale exome sequencing study implicates both developmental and functional changes in the neurobiology of autism. *Cell* 180 568.e23–584.e23. 31981491 10.1016/j.cell.2019.12.036 PMC7250485

[B83] Shin H. Y. Pfaff K. L. Davidow L. S. Sun C. Uozumi T. Yanagawa F. (2018). Using automated live cell imaging to reveal early changes during human motor neuron degeneration. *eNeuro* 5:ENEURO.0001-18.2018. 10.1523/ENEURO.0001-18.2018 PMC6026021 29971247

[B84] Song Y. Jia Z. C. Chen J. Y. Hu J. X. Zhang L. S. (2014). Toxic effects of atrazine on reproductive system of male rats. *Biomed. Environ. Sci.* 27 281–288. 24758756 10.3967/bes2014.050

[B85] Suzuki S. Akamatsu W. Kisa F. Sone T. Ishikawa K.-I. Kuzumaki N. (2017). Efficient induction of dopaminergic neuron differentiation from induced pluripotent stem cells reveals impaired mitophagy in PARK2 neurons. *Biochem. Biophys. Res. Commun.* 483 88–93. 10.1016/j.bbrc.2016.12.188 28057485

[B86] Takahashi K. Yamanaka S. (2006). Induction of pluripotent stem cells from mouse embryonic and adult fibroblast cultures by defined factors. *Cell* 126 663–676. 10.1016/j.cell.2006.07.024 16904174

[B87] Thijssen E. H. La Joie R. Wolf A. Strom A. Wang P. Iaccarino L. (2020). Diagnostic value of plasma phosphorylated tau181 in Alzheimer’s disease and frontotemporal lobar degeneration. *Nat. Med.* 26 387–397. 32123386 10.1038/s41591-020-0762-2 PMC7101073

[B88] Thiruchelvam M. Richfield E. K. Goodman B. M. Baggs R. B. Cory-Slechta D. A. (2002). Developmental exposure to the pesticides paraquat and maneb and the Parkinson’s Disease phenotype. *NeuroToxicology* 23 621–633. 10.1016/s0161-813x(02)00092-x 12428734

[B89] Tsien R. Y. Pozzan T. Rink T. J. (1982). Calcium homeostasis in intact lymphocytes: cytoplasmic free calcium monitored with a new., intracellularly trapped fluorescent indicator. *J. Cell Biol.* 94 325–334. 10.1083/jcb.94.2.325 6980885 PMC2112871

[B90] Vallerga C. L. Zhang F. Fowdar J. McRae A. F. Qi T. Nabais M. F. (2020). Analysis of DNA methylation associates the cystine–glutamate antiporter SLC7A11 with risk of Parkinson’s disease. *Nat. Commun.* 11:1238. 10.1038/s41467-020-15065-7 PMC7060318 32144264

[B91] Velasco S. Kedaigle A. J. Simmons S. K. Nash A. Rocha M. Quadrato G. (2019). Individual brain organoids reproducibly form cell diversity of the human cerebral cortex. *Nature* 570 523–527. 10.1038/s41586-019-1289-x 31168097 PMC6906116

[B92] Wang Y. Shi M. Chung K. A. Zabetian C. P. Leverenz J. B. Berg D. (2012). Phosphorylated α-synuclein in Parkinson’s disease. *Sci. Transl. Med.* 4:121ra120. 10.1126/scitranslmed.3002566 PMC3302662 22344688

[B93] Wang Z. Zheng Y. Zhao B. Zhang Y. Liu Z. Xu J. (2015). Human metabolic responses to chronic environmental polycyclic aromatic hydrocarbon exposure by a metabolomic approach. *J. Proteome Res.* 14 2583–2593. 10.1021/acs.jproteome.5b00134 25990285

[B94] Watras C. J. Back R. C. Halvorsen S. Hudson R. J. M. Morrison K. A. Wente S. P. (1998). Bioaccumulation of mercury in pelagic freshwater food webs. *Sci. Total Environ.* 219 183–208. 10.1016/s0048-9697(98)00228-9 9802248

[B95] Weber G. J. Sepúlveda M. S. Peterson S. M. Lewis S. S. Freeman J. L. (2013). Transcriptome alterations following developmental atrazine exposure in zebrafish are associated with disruption of neuroendocrine and reproductive system function., cell cycle., and carcinogenesis. *Toxicol. Sci.* 132 458–466. 10.1093/toxsci/kft017 23358194 PMC3595526

[B96] Wirbisky S. E. Weber G. J. Sepúlveda M. S. Lin T.-L. Jannasch A. S. Freeman J. L. (2016). An embryonic atrazine exposure results in reproductive dysfunction in adult zebrafish and morphological alterations in their offspring. *Sci. Rep.* 6:21337. 10.1038/srep21337 PMC4759560 26891955

[B97] Wirbisky-Hershberger S. E. Sanchez O. F. Horzmann K. A. Thanki D. Yuan C. L. Freeman J. L. (2017). Atrazine exposure decreases the activity of DNMTs., global DNA methylation levels., and dnmt expression. *Food Chem. Toxicol.* 109 727–734. 10.1016/j.fct.2017.08.041 28859886 PMC5656531

[B98] Workman A. D. Charvet C. J. Clancy B. Darlington R. B. Finlay B. L. (2013). Modeling transformations of neurodevelopmental sequences across Mammalian species. *J. Neurosci.* 33 7368–7383. 10.1523/jneurosci.5746-12.2013 23616543 PMC3928428

[B99] Yang H. H. St-Pierre F. Sun X. Ding X. Lin M. Z. Clandinin T. R. (2016). Subcellular imaging of voltage and calcium signals reveals neural processing in vivo. *Cell* 166 245–257. 10.1016/j.cell.2016.05.031 27264607 PMC5606228

[B100] Yang Y. Liu N. He Y. Liu Y. Ge L. Zou L. (2018). Improved calcium sensor GCaMP-X overcomes the calcium channel perturbations induced by the calmodulin in GCaMP. *Nat. Commun.* 9:1504. 10.1038/s41467-018-03719-6 PMC5904127 29666364

